# Swelling-Based Distributed Chemical Sensing with Standard Acrylate Coated Optical Fibers

**DOI:** 10.3390/s21030718

**Published:** 2021-01-21

**Authors:** Sina Sedighi, Marcelo A. Soto, Alin Jderu, Dorel Dorobantu, Marius Enachescu, Dominik Ziegler

**Affiliations:** 1 NanoPRO START S.R.L., Oltenitei, No. 388, District 4, 041337 Bucharest, Romania; sina.sedig@gmail.com (S.S.); alin.jderu@cssnt-upb.ro (A.J.); dorel.dorobantu@cssnt-upb.ro (D.D.); 2 Department of Electronic Engineering, Universidad Técnica Federico Santa María, 2390123 Valparaíso, Chile; marcelo.sotoh@usm.cl; 3 Center for Surface Science and Nanotechnology (CSSNT), University Politehnica Bucharest, 060042 Bucharest, Romania; marius.enachescu@cssnt-upb.ro; 4 Academy of Romanian Scientists, 54 Splaiul Independentei, 050094 Bucharest, Romania

**Keywords:** fiber optics sensors, distributed optical fiber sensor, optical frequency-domain reflectometry, chemical sensing

## Abstract

Distributed chemical sensing is demonstrated using standard acrylate coated optical fibers. Swelling of the polymer coating induces strain in the fiber’s silica core provoking a local refractive index change which is detectable all along an optical fiber by advanced distributed sensing techniques. Thermal effects can be discriminated from strain using uncoated fiber segments, leading to more accurate strain readings. The concept has been validated by measuring strain responses of various aqueous and organic solvents and different chain length alkanes and blends thereof. Although demonstrated on a short range of two meters using optical frequency-domain reflectometry, the technique can be applied to many kilometer-long fiber installations. Low-cost and insensitive to corrosion and electromagnetic radiation, along with the possibility to interrogate thousands of independent measurement points along a single optical fiber, this novel technique is likely to find applications in environmental monitoring, food analysis, agriculture, water quality monitoring, or medical diagnostics.

## 1. Introduction

With nearly two billion kilometers deployed around the world, optical fibers are the backbone of today’s telecommunications networks. Their high reliability, low losses, and low cost, together with immunity to electromagnetic fields and remarkable light guidance, make optical fibers hold great promise for sensing applications [1]. Among the many different sensing approaches that have evolved over the last decades, distributed optical fiber sensors have gained attention due to their unique ability to provide longitudinally distributed measurements of physical variables, such as temperature or strain [2].

Using the natural response of scattering processes (such as Brillouin, Raman, or Rayleigh scattering) to environmental changes, distinct distributed optical fiber sensors [3,4,5] have been exploited to monitor a variety of physical properties. The interrogating systems typically use optical frequency-domain reflectometry (OFDR) or optical time-domain reflectometry (OTDR) to obtain spatially resolved information about the measurand. Among these techniques, OTDR is commonly used over long distances (e.g., tens of kilometers); however, it offers limited spatial resolution. On the other hand, OFDR achieves very high spatial resolution (millimeter length scale), yet the sensing distance is limited to a few tens of meters. These technologies primarily measure temperature and strain changes [2]; however, there has been a recent interest in expanding the number of physical variables that a distributed fiber sensor can measure. Non-standard optical fibers, such as microstructured fibers, multicore fibers, or fibers with special coatings, are used to convert the environmental variable of interest into strain or temperature changes in the optical fiber, enabling the distributed measurement of hydrostatic pressure [6,7], humidity [8,9,10], electric current [11], and electromagnetic field [12,13].

In the field of chemical sensing, optical fiber approaches have been investigated for many years [14]. Since silica optical fibers are chemically inert, fiber-optic based chemical sensing commonly requires converting the analyte information into changes of detectable quantities such as temperature or strain [14,15,16]. However, most of the chemical sensing approaches only probe substances at the end of the fiber or at few discrete locations, where the light is purposefully guided into a sensing element [15,16]. For distributed sensing, specialty fibers such as plastic or microstructured optical fibers are typically exploited [17,18], to allow for instance highly sensitive distributed gas sensing [19,20] or distributed humidity sensors [10]. Distributed gas sensing can be obtained by exploiting photothermal spectroscopy in microstructured fibers, in which the laser light is tuned on a gas molecular absorption line to increase the gas temperature, which is then measured by a distributed temperature sensing technique such as Rayleigh-based OTDR [19,20]. Using water-swellable polymers to coat silica fibers and reflectometry techniques, distributed humidity sensors have been reported [21]. In such a case, the mechanism behind distributed humidity sensing is based on the distributed measurements of the axial strain induced on the optical fiber by the expansion that experiences a hygroscopic fiber coating when exposed to water molecules [21]. Making use of other chemically sensitive swellable polymers, different chemical species can be detected by measuring the swelling-induced strain in the fiber. However, this approach has been mostly exploited for discrete point sensing, for instance, based on fiber Bragg gratings [22]. Only a few similar approaches have been reported for distributed sensing, being essentially used for distributed humidity sensing [8,9] and distributed pH sensing [23]. Recently, guided acoustic-wave Brillouin scattering has been explored for distributed sensing of external liquid analytes based on acoustic impedance mismatch in the interface between the optical fiber and the liquid solution [24,25,26].

This paper exploits OFDR interrogation to experimentally validate a distributed chemical sensor based on an unmodified commercial optical fiber with acrylate coating. To the best of our knowledge, this is the first report of detecting the presence of chemicals in a distributed manner using the swelling response of a conventional acrylate coated optical fiber. While a silica optical fiber is chemically inert, the work proposed here exploits the chemical response of conventional acrylate coating in optical fibers. The observed swelling of acrylate coatings in presence of water, ethanol, isopropanol alcohol, acetone, and alkanes is demonstrated here. Although a similar approach has been already demonstrated for distributed humidity sensing owing to the hygroscopic feature of acrylate coatings [8,9], to the best of our knowledge, the principle has never been exploited for distributed sensing of chemical solvents. Results demonstrate that the swelling capability of the acrylate coating to the analyzed solvents provides measurable fiber strain responses. Exploiting the distributed features of the measurements and the use of uncoated optical fiber sections, the chemical sensing response is discriminated from temperature responses due to excess enthalpies of mixing. This way, while uncoated fiber sections only measure temperature changes, coated section measure both temperature and swelling strain. Using the temperature measurements, the swelling strain in the coated sections could be estimated. Moreover, unlike existing chemical sensors that rely on power loss, the technique reported here combines OTDR interrogation methods with swelling-induced strain changes and could enable distributed sensing to reach very long distances with thousands of independent sensing points along a single optical fiber. The proposed approach could enable new applications of distributed chemical sensing, for instance in leak detection in pipelines, monitoring of water quality or monitoring of supply lines for chemical, medical, food, or pharmaceutical industries.

## 2. Materials and Methods

### 2.1. Chemical Sensing-Based on Swelling Mechanism

**Figure 1:**
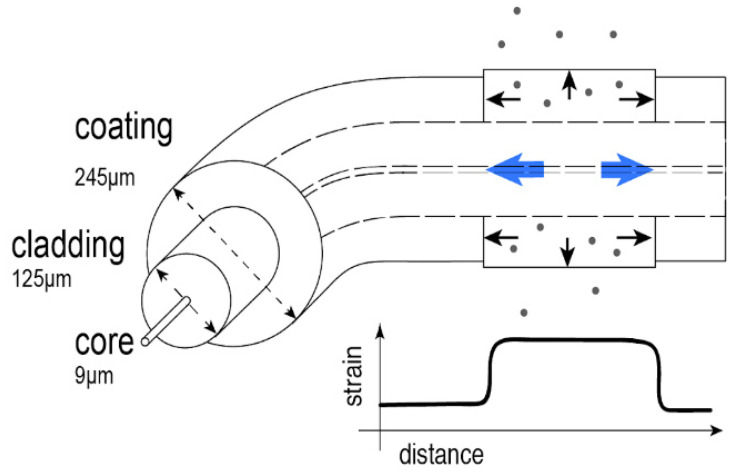
Distributed chemical sensing principle using conventional acrylate coated optical fibers. Gray dots represent an analyte in solution that is penetrating a section of the permeable coating. As the coating swells, it applies a local axial tensile strain on the fiber.

Figure 1 illustrates the chemical sensing principle. An optical fiber section is exposed to a chemical substance and penetration of that chemical into the coating induces a swelling that strains the optical fiber itself. While the optical properties of the fiber core and cladding make efficient light transmission possible along the optical waveguide, the coating material itself is not optically active. Traditionally, the coating provides mechanical strength and protects the fragile glass fiber to improve reliability. The polymer coating materials typically used in conventional optical fibers are optimized for slow aging and high temperature resistance. When exposed to a chemical, such as a chemical solvent can penetrate the coating, thus expanding its dimensions [27]. The axial expansion induces strain onto the fiber core and cladding. To perform distributed chemical sensing, such swelling strain can be measured with a distributed sensing technique. While Brillouin-based approaches [4] would be preferred to monitor the presence of chemicals over very long fibers, their metric spatial resolution and long measurement times make Rayleigh-based approaches [5] much more attractive due to the higher spatial and strain resolutions that can be obtained. In this case, Rayleigh-based OFDR [28] is used to attain millimetric spatial resolution with interrogation rates of a few Hz.

### 2.2. Optical Frequency-Domain Reflectometry (OFDR)

**Figure 2:**
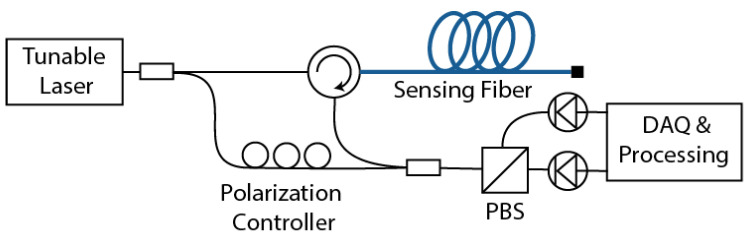
Schematic of a conventional optical frequency-domain reflectometry (OFDR) system implementation.

Distributed optical fiber sensing based on Rayleigh scattering makes use of the interference pattern generated by the coherent backscattered Rayleigh light along an optical fiber [5,28]. Figure 2 shows a general basic schematic of the implementation of an OFDR sensor. The system uses the light of a tunable laser, whose wavelength is linearly swept over time, and then launched through a circulator into a sensing fiber. A low-power copy of the laser light is obtained as a local oscillator using an optical splitter. The coherent Rayleigh light backscattered from the sensing fiber is collected by the circulator and combined with the local oscillator through a coupler. To avoid polarizations fading in the measurements, a polarization controller is normally used to evenly split the power of the local oscillator into two orthogonal states of polarization matching the axes of a polarization beam splitter (PBS), which is used to separate orthogonal polarizations into two independent photodetectors. A data acquisition system (DAQ) together with a processing unit are then used to acquire the photo-detected signals and process them to extract the useful information.

The signal processing required to extract the Rayleigh optical phase, containing information about strain and temperature changes in the fiber, involves the use of Fourier transform and spectral correlations [28]. The spatial resolution of the sensor is determined by the frequency sweeping range of the laser, typically resulting in millimetric resolution. The measurement procedure requires the acquisition of a first reference Rayleigh signal, whose local spectrum at each fiber location is then compared through cross-correlation with the Rayleigh spectrum obtained by the actual (real-time) measurements. This cross-correlation results in a Rayleigh frequency shift (related to the optical phase shift), which is proportional to temperature and strain changes in the fiber. The fiber’s refractive index changes with both temperature and strain; and therefore, many techniques have been proposed in the literature to discriminate temperature from strain in Rayleigh-based sensors [29,30,31], including some involving complex setups. In this work, a partially coated fiber is used to discriminate temperature from strain. Coated fiber sections measure both temperature changes (e.g., resulting from the heat of mixing in the chemical reaction) and swelling-induced strain applied by the expanded coating onto the sensing fiber. Uncoated sections only measure temperature changes. Using this temperature information, the swelling strain in the coated sections could be estimated.

## 3. Results

### 3.1. Distributed Measurements of Water-Alcohol Mixtures

The distributed chemical sensing capabilities of the proposed system are verified using a 2 m long standard optical fiber with acrylate coating, which is interrogated by a commercial OFDR system (ODiSI-B by Luna Technologies Inc., Roanoke, VA, USA) using a spatial resolution of 1.25 mm and 4 Hz temporal sampling rate. Three different optical fiber segments with lengths of about 25 cm each are immersed into three consecutive solvent baths. First, an OFDR baseline strain recording is taken with the entire fiber in air. After filling the three baths with 100 mL of water, the strain response of the sensing fiber is monitored over time at each fiber location. During the experiment, the concentrations of the organic solvents are increased stepwise. Each bath contains water and one solvent: Ethanol, IPA, or acetone. A swift exchange of liquids is facilitated using a syringe to remove a fixed amount of the existing liquid from the bath and quickly replenishing it with the required amount of pure solvent to reach target concentrations. Since ethanol, IPA, and acetone are polar solvents that are well miscible in water, no stirring is required to achieve uniform concentrations throughout the baths.

**Figure 3:**
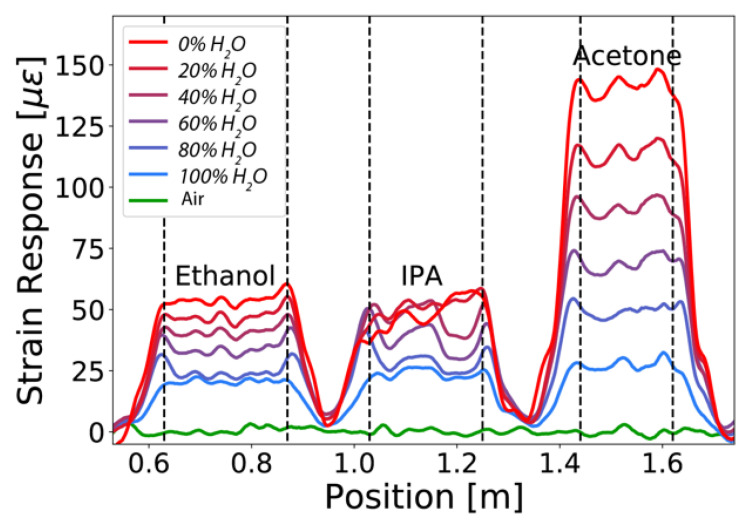
Distributed swelling strain response measured in three different baths containing ethanol, isopropyl alcohol (IPA), and acetone. The volume percent of the water content is varied as indicated in the legend. The dashed vertical lines indicate the boundaries of the segments of the fiber which are fully immersed.

Figure 3 demonstrates the capability of the sensor to simultaneously measure responses to different organic solvents in a distributed way, showing the swelling-induced strain as a function of the fiber position. The figure shows the strain response profiles measured upon stepwise increases of the concentration at 17 min intervals. As the acquisition rate (4 Hz) faster than what is required to capture the slow dynamics of the strain evolution, 100 traces are averaged over time for better visualization.

A generally monotonous increase of the induced strain is verified with increasing concentrations of ethanol, IPA, and acetone. The only exception is for the 100% IPA solution, for which a decrease of strain is observed between 1.0 m and 1.1 m. Partial dissolution of the polymer coating or eventual delamination from the cladding could explain this observation. This effect is not observed for ethanol and acetone even after a prolonged exposure during the experiment. While the overall strain for IPA and ethanol are comparable, the swelling strain for acetone is about three times higher. This is likely due to the aprotic nature of acetone, which makes it a better solvent for acrylic materials [32]. The same sensor response is observed when repeating the experiment under the same conditions, confirming that the sensor was not degrading in ethanol and acetone. The high spatial resolution of the sensor allows us to observe small strain variations along the immersed fiber sections. The average strain grows with increasing solvent concentration, and the amplitude in strain variation increases with higher concentrations. Thickness variations of the coating along the fiber are likely to be the origin of these variations and could be easily compensated by a local fiber response calibration.

**Figure 4:**
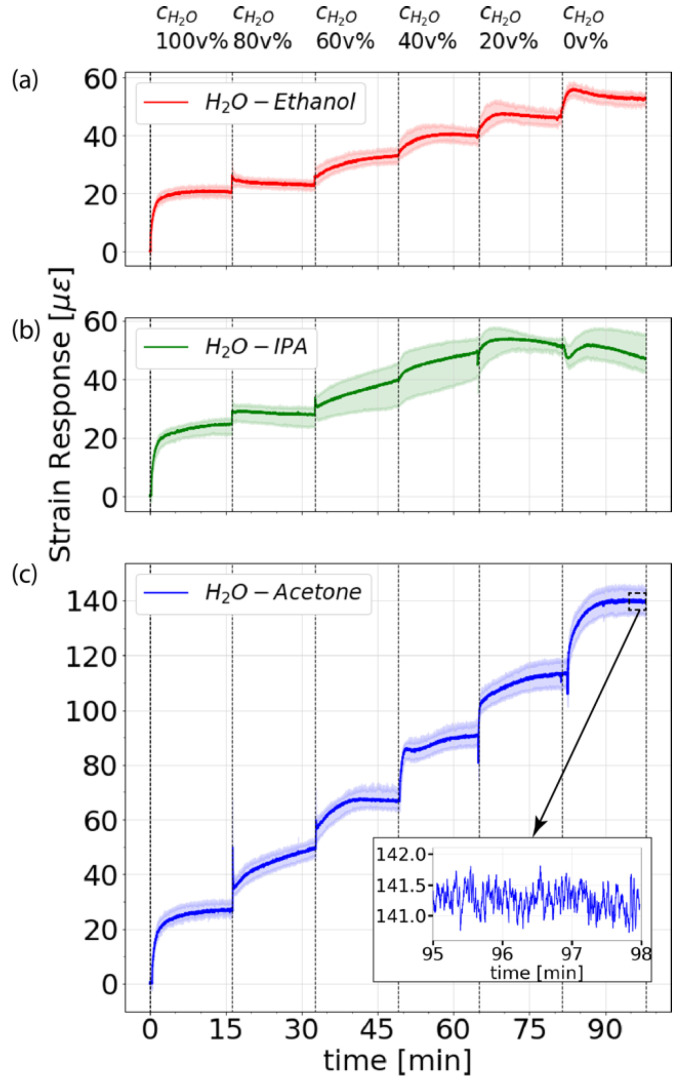
Temporal evolution of the swelling-induced strain for the three baths containing (**a**) ethanol, (**b**) IPA, and (**c**) acetone in water. The solid lines show the average values for the immersed sections. The colored regions depict the entire strain range measured at the different fiber positions. The inset graph shows the last three minutes of the strain response for acetone, highlighting the low measurement noise of the system.

Figure 4 shows the temporal evolution of the measured swelling-induced strain from the moment of exposing the fiber segments to water. The hygroscopic coating swells as water molecules migrate inward. The strain evolution corresponds to the average value (solid lines) measured within the fiber sections immersed in the baths as indicated by the vertical dashed lines in Figure 3. The colored regions depict the entire strain range measured at the different fiber positions. As solvent concentration increases, a peak appears in the measured strain evolution. As described in Section 3.2, these peaks result from rapid thermal changes caused by the heat of mixing that occurs when solvents are added to water. The thermal increase only affects the measurements during the first 1–2 min after mixing. Stable swelling-induced strain measurements can be obtained after several minutes. Results indicate a linear increase in the measured swelling-induced strain with the solvent volumetric concentration, resulting in a sensitivity of about 0.33 µε/v%, 0.40 µε/v%, and 1.15 µε/v% for ethanol, IPA and acetone, respectively. Considering the fiber’s strain sensitivity (−150 MHz/µε), the Rayleigh frequency sensitivities to concentrations are −49.5 MHz/v%, −60.0 MHz/v% and −172.5 MHz/v% for each of the respective solvents.

The inset of Figure 4 illustrates the measured strain over the last minutes of the experiment, where a flat strain response is obtained, and a standard deviation of 0.196 µε is verified. This value allows us to estimate the detection limit in terms of an actual percentual volumetric concentration that the sensor can detect. Detection limits of 0.59 v%, 0.49 v%, and 0.17 v% are verified for ethanol, IPA, and acetone, respectively.

### 3.2. Monitoring Thermal Effects due to Excess Enthalpy of Mixing of Acetone and Water

When mixing water with any of the analyzed organic solvents, a temperature change results from the released enthalpy of mixing [33]. The temperature change occurs simultaneously with the swelling strain and is responsible for the peaks appearing in Figure 4. To discriminate temperature artifacts from strain, an optical fiber with a partially stripped coating is used. Fiber sections with no coating monitor temperature changes resulting from the enthalpy of mixing, while coated sections simultaneously measure strain and thermal changes. With both measurements, a temperature-compensated reading could be acquired to unambiguously interpret data from strain-induced changes. To perform these measurements, the fiber coating is removed from 25 cm long sections. Although chemical dissolution [34] and laser ablation methods [35] can be used for this process, manual stripping the coating from short fiber segments is here performed using a conventional fiber stripping tool. Optical reflectometry is performed to confirm the fiber integrity following coating removal.

**Figure 5:**
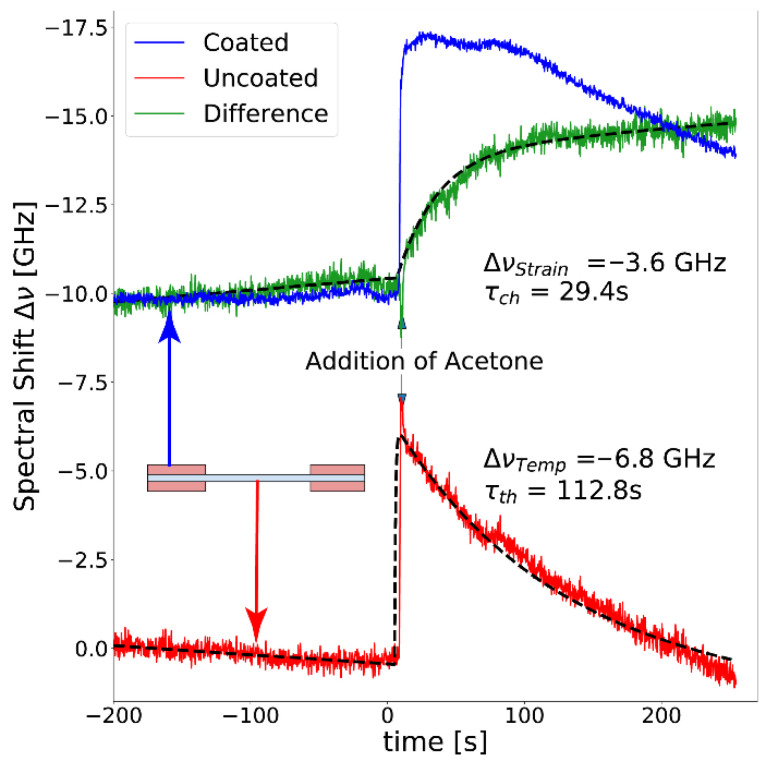
Rayleigh frequency shift measured from coated (blue line) and uncoated (red line) fiber sections, allowing to discriminate the temperature effects due to the water-acetone heating of mixing from swelling strain. The green line shows the temperature-compensated swelling strain built-up from the exposure of the fiber coating to a 17% molar acetone-water mixture.

Figure 5 shows the Rayleigh spectral shift measured in the coated (blue curve) and uncoated (red curve) immersed sections. The Rayleigh frequency shift Δν resulting from the temperature increase upon mixing acetone and water due to heat of mixing can be clearly measured in the uncoated fiber section. The measurement in the coated fiber section represents the combined effect of temperature and swelling-induced strain changes. Subtracting the thermal evolution from the coated fiber response, the pure chemical response of the fiber to solvent concentration is obtained, as shown by the green curve in Figure 5.

Results show a maximum Rayleigh spectral shift of −6.8 GHz, which based on the temperature sensitivity of the fiber (−1.57 GHz/K), represents a sudden temperature change of 4.3 K. For a thermally isolated system with zero heat dissipation, the maximum excess temperature due to heat of mixing when adding acetone up to a 17% molar concentration to water is estimated to be 7.2 K (based on the water-acetone enthalpy of mixing described in the literature [33]). The values are in reasonable agreement considering the various heat losses (conduction, convection, evaporative cooling) in the system, which can be clearly observed in the rapid thermal exponential decay (τ = 112.8 s). On the other hand, a Rayleigh spectral shift of −3.6 GHz is obtained from the temperature-compensated measurement of the swelling-induced strain, which corresponds to a strain of 24 µε.

### 3.3. Measuring n-Alkanes Blends

**Figure 6:**
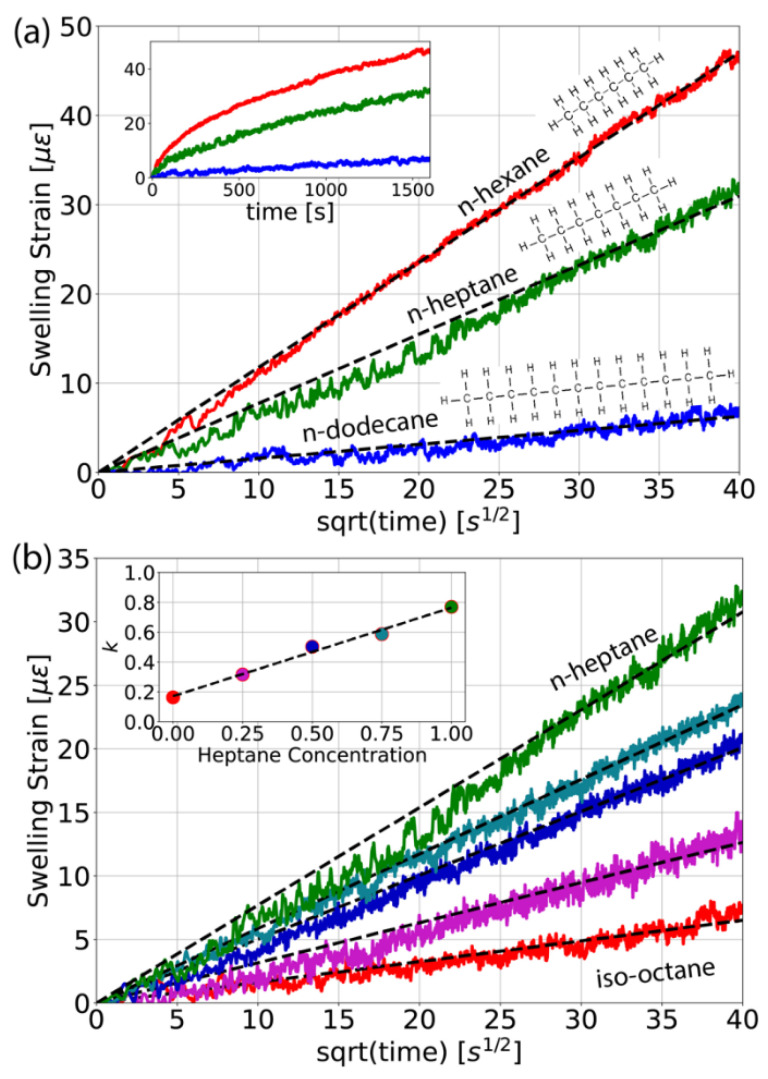
Swelling strain for alkanes plotted against the square root of time. (**a**) Different chain length; the inset shows the same data plotted against time. (**b**) Different concentrations of n-heptane in iso-octane from 0% to 100% heptane concentration with steps of 25%. The inset shows that the diffusion constants increase linearly with the concentration of n-heptane in iso-octane.

The ability of standard acrylate coated optical fibers to discriminate alkanes with different chain length, as well as blends thereof is also characterized. Alkanes are organic compounds that consist entirely of single-bonded carbon and hydrogen atoms without any other functional groups [36]. Alkane molecules with 5–17 carbon atoms are liquid at room temperature and key components found in gasoline. Figure 6a shows the temporal evolution of the longitudinally averaged swelling strain measured by the proposed sensing approach, for different concentrations of n-hexane (C_6_H_14_), n-heptane (C_7_H_16_), and n-dodecane (C_12_H_26_). Results indicate that shorter chain length alkane molecules result in stronger swelling effects. The length difference of only one carbon atom between n-heptane and n-hexane can easily be discerned. Penetration kinetics of solvents in polymethyl methacrylate can exhibit a variety of mechanisms [37]. The most common mechanism is a diffusion driven (Fickian) process. The inset in Figure 6a shows the swelling-induced strain as a function of time for three different chain length alkanes. Swelling kinetics in polymers can have complex dynamics with relaxation times that can be minutes to days. Here, even after 30 min the strain did not reach a steady state. A descriptive way to quantify diffusion kinetics is the power law *X = k t^m^* where *X* is the distance covered by the solvent front at time *t*, and *k* is a constant of the system and *m* the exponent, for Fickian kinetics *m* = 0.5 [38]. From the slope of the data plotted against the square root of time in Figure 6a, the constant *k* is estimated, which results in 1.18, 0.77, and 0.16 for n-hexane, n-heptane and n-dodecane, respectively. Results suggest that for the time frame of the measurements of 30 min the swelling mechanism is predominantly through a Fickian transport process. It is interesting to note that the first few seconds already provide the characteristic information regarding diffusion properties of the polymer-solvent pair. Figure 6b demonstrates that dilution of n-heptane in iso-octane linearly decreases the swelling strain with concentration. The inset shows that the resulting diffusion constants increase linearly for the five different concentrations measured.

## 4. Conclusions

In this paper the response of a standard optical fiber with acrylate coating to organic solvents has been characterized in combination to OFDR interrogation for distributed chemical sensing. The sensing technique is based on the detection of strain changes induced by the expansion of the standard acrylate coating in response to analytes including ethanol, isopropanol alcohol, and acetone. Swelling strain increases with the solvent concentration which demonstrates that conventional, unmodified optical fibers with acrylate coatings possess the potential to be used for distributed chemical sensing. Detection limits of less than one volume percent have been verified when using millimetric spatial resolution. Further research could develop advanced coatings to target specific molecules to deliver both superior selectivity and even lower detection levels than the simple acrylic coating tested here. A partially coated fiber has also been demonstrated to discriminate swelling strain from temperature changes resulting from the heat of mixing of the solvent and water. Distributed sensing can enable the development of true fingerprinting techniques, where multiple sensors with ideally orthogonal responses can be used to identify chemicals. To this end, short segments of the fiber could be recoated with specific polymer that show a different response to a target chemical. Development of recoating techniques is part of ongoing research. While the experiments here were performed on a short optical fiber using OFDR, the proposed chemical sensing approach could be applicable to fiber sensing ranges exceeding 100 km, using Brillouin or Rayleigh time-domain interrogation techniques. Such distributed chemical sensing techniques have applications in water quality monitoring, hydrocarbon concentration in gasoline, leak detection of gas or oil pipelines or any type of supply lines for chemical, medical, food or pharmaceutical industries. The fact that a single interrogator unit monitors the presence of chemicals at thousands of locations along the fiber might help to monitor the reactions of large assays where many tests are to be performed in parallel.

## 5. Patents

Measurement method and sensor device for chemical detection using optical fibers, Patent filed with “State Office for Inventions and Trademarks”-Romania, nr. A/00744, 18 November 2020.
